# Characterized the Adipogenic Capacity of Adipose-Derived Stem Cell, Extracellular Matrix, and Microenvironment With Fat Components Grafting

**DOI:** 10.3389/fcell.2021.723057

**Published:** 2021-09-20

**Authors:** Wenqing Jiang, Junrong Cai, Jingyan Guan, Yunjun Liao, Feng Lu, Jingjing Ma, Jianhua Gao, Yuteng Zhang

**Affiliations:** ^1^Department of Plastic and Cosmetic Surgery, Nanfang Hospital, Southern Medical University, Guangzhou, China; ^2^Department of Plastic Surgery, Sir Run Run Shaw Hospital, School of Medicine, Zhejiang University, Hangzhou, China

**Keywords:** fat grafting, adipogenesis, adipose-derived stem cell, extracellular matrix, microenvironment

## Abstract

**Background:** Autologous fat grafting has been a widely used technique; however, the role of adipose-derived stem cells (ASCs), extracellular matrix (ECM), and microenvironment in fat regeneration are not fully understood.

**Methods:** Lipoaspirates were obtained and processed by inter-syringe shifting to remove adipocytes, yielding an adipocyte-free fat (Aff). Aff was then exposed to lethal dose of radiation to obtain decellularized fat (Df). To further remove microenvironment, Df was rinsed with phosphate-buffered saline (PBS) yielding rinsed decellularized fat (Rdf). Green fluorescent protein (GFP) lentivirus (LV-GFP)-transfected ASCs were added to Df to generate cell-recombinant decellularized fat (Crdf). Grafts were transplanted subcutaneously into nude mice and harvested over 3 months.

**Results:** Removal of adipocytes (Aff) didn’t compromise the retention of fat grafts, while additional removal of stromal vascular fraction (SVF) cells (Df) and microenvironment (Rdf) resulted in poor retention by day 90 (Aff, 82 ± 7.1% vs. Df, 28 ± 6.3%; *p* < 0.05; vs. Rdf, 5 ± 1.2%; *p* < 0.05). Addition of ASCs to Df (Crdf) partially restored its regenerative potential. Aff and Crdf exhibited rapid angiogenesis and M2-polarized macrophages infiltration, in contrast to impaired angiogenesis and M1-polarized inflammatory pattern in Df. GFP + ASCs participated in angiogenesis and displayed a phenotype of endothelial cells in Crdf.

**Conclusion:** Adipose ECM and microenvironment have the capacity to stimulate early adipogenesis while ECM alone cannot induce adipogenesis *in vivo*. By directly differentiating into endothelial cells and regulating macrophage polarization, ASCs coordinate early adipogenesis with angiogenesis and tissue remodeling, leading to better long-term retention and greater tissue integrity.

## Introduction

Autologous fat grafting has increasingly been applied to promote volume augmentation and facilitate tissue regeneration, and progress in this field has been rapid (Strong et al., 2015; Khouri, 2017). However, the retention rate of mass volume engraftment remains suboptimal, and complications are unpredictable (Yoshimura and Coleman, 2015; Groen et al., 2016; Lv et al., 2020). Due to the interdependent dynamic changes of various components during grafting, the technique for ideal fat grafting remains controversial.

The “cell replacement theory” describes three different outcomes from the periphery to the center of the graft: survival, regeneration, and necrosis. As transplant volume increases, the most central adipocytes in the graft undergo ischemic necrosis (Kato et al., 2014; Mashiko and Yoshimura, 2015). The necrotic and regenerative ratios of the transplanted tissue will determine the final tissue retention and morphotype (Dong et al., 2015). To improve regenerative outcomes, it is essential to elucidate the detailed mechanisms underlying the components of fat grafts.

Adipocytes constitute about 90% of graft volume (Rotondo et al., 2016). Severe ischemia and hypoxia after avascular grafting limit the function of these cells, and most undergo apoptosis before re-vascularization. By contrast, nucleated stromal cells, especially adipose-derived stem cells (ASCs), are capable of surviving extreme circumstances and actively contributing to tissue regeneration (Suga et al., 2010). Studies of ASCs have revealed that these cells have a variety of functions, including enhanced proliferation, migration, and paracrine angiogenic cytokines, which make them more likely to survive the hypoxic insult of transplantation and expedite vascularization (Kang et al., 2014). In conjunction with supplemental ASCs or SVF, the cell-assisted lipotransfer (CAL) technique has had promising effects on retention and graft quality, yielding considerably higher residual volumes and less necrotic tissue (Kolle et al., 2013; Toyserkani et al., 2016; Zhou et al., 2016). Together, these results suggest that ASCs have tremendous potential for promoting fat regeneration.

Meanwhile, the extracellular matrix (ECM) and microenvironment of adipose tissue also play an essential role in fat regeneration (Flynn, 2010; Wu et al., 2012; Sano et al., 2014). Recent studies illustrated the adipoinduction potential and prospective volume retention rate of Allograft Adipose Matrix (AAM) (Giatsidis et al., 2019). Kokai et al. (2019) applied AAM allogenetically in a clinical trial; the material achieved a 44 ± 16% retention rate after 24 weeks *in vivo* by inducing host-derived vascular invasion and adipose regeneration (Kokai et al., 2019). This study showed for the first time that AAM promotes adipose regeneration. Moreover, in a murine model, by significantly improving recellularization and angiogenesis, ASC-seeded decellularized adipose matrix improved long-term retention after transplantation (Turner et al., 2012; Han et al., 2015; Trivanovic et al., 2018). However, the role of each component of fat tissue in fat grafting is still unclear. Elucidating the relationship among ASCs, ECM and microenvironment will provide insight into the essential elements of adipose regeneration and contribute to the development of adipose transplantation procedures.

In this study, we used different mechanical processes to alter the components in fat tissue and test the relevant fat products’ retention rate. Inter-syringe shifting and centrifugation were used to remove most adipocytes, but preserved the SVF cells and original ECM (Yao et al., 2017). By further eliminating the SVF cells in the fat tissue, radiation is applied to the fat tissue and resulted in a cell-free adipose tissue which is mainly comprised of ECM and the soluble proteins (microenvironment) (Jonathan et al., 1999; Favaudon et al., 2014). This product is furthered rinsed to remove the soluble proteins to explore the role of microenvironment in fat tissue. By removing different components in fat tissue step by step, it is possible to assess the orchestration among ASCs, ECM, and microenvironment after fat grafting.

## Materials and Methods

### Preparation of Adipocyte-Free Fat (Aff), Decellularized Fat (Df), and Rinsed Decellularized Fat (Rdf)

Human lipoaspirates were obtained using standard Coleman methods from 11 healthy women, with mean ± SD age of 33.4 ± 6.3 years and mean ± SD body mass index of 23.2 ± 1.9 kg/m2. To remove adipocytes, fat was mechanically emulsified by shifting between two regular syringes. The emulsified fat was then centrifuged to remove the upper layer of oil. The lower layer was used as Aff. To remove SVF cells, the prepared Aff was subjected to lethal radiation at a rate of 300 cGy/min for 40 min using the 6 MV photon beam of the Varian 23EX linear accelerator (Varian Medical Systems, Palo Alto, CA, United States) and the resulted product was termed as Df. To remove the soluble proteins, the prepared Df was rinsed three times with PBS and centrifuged at 1,200 *g* for 3 min to obtain Rdf.

### Scanning Electron Microscopy

Samples were fixed with 2% glutaraldehyde in 0.1 M phosphate buffer, postfixed in 1% osmium tetroxide in the same buffer for 1 h, dehydrated in increasing concentrations of acetone, critical-point dried, fixed to stubs with colloidal silver, sputtered with gold using a MED 010 coater, and examined under an S-3000N scanning electron microscope (Hitachi, Ltd., Tokyo, Japan).

### Isolation and Culture of Adipose-Derived Stem Cells

Adipocyte-free fat and decellularized fat were washed with PBS and digested with 0.075% type I collagenase (Sigma-Aldrich, St. Louis, MO, United States). After inactivation of collagenase activity, the cell suspension was filtered through a 40 μm cell strainer (BD Biosciences, San Jose, CA, United States) and centrifuged at 1,200 *g* for 3 min. Red blood cell lysis buffer (Leagene Biotech, Beijing, China) was used to remove erythrocytes. The cells were then collected by removal of supernatant, and then resuspended in human adipose-derived mesenchymal stem cell complete medium (Cyagen, Santa Clara, CA, United States). Cell viability was investigated 72 h after culture using the Live/Dead assay kit (Invitrogen, Carlsbad, CA, United States).

### Recombination of Green Fluorescent Protein-Transfected Adipose-Derived Stem Cells to Decellularized Fat (Cell-Recombinant Decellularized Fat)

LV vectors containing green fluorescent protein (GFP) were constructed and designated as LV-GFP (GeneChem, Shanghai, China). ASCs isolated from Aff were transfected with LV-GFP at a multiplicity of infection (MOI) of 80 in the presence of Polybrene (6 μg/ml) for 8 h. After 12 h, the supernatant was replaced with culture medium. Crdf was generated by mixture of 5 × 105 transfected cells per 1 ml Df by gentle shaking.

### Graft Model in Nude Mice

All animal experiments were approved by the Nanfang Hospital Institutional Animal Care and Use Committee and performed according to the guidelines of the National Health and Medical Research Council (People’s Republic of China). Nude mice (aged 6–8 weeks) were housed in individual cages with a 12 h light/dark cycle and provided with standard food and water *ad libitum*. Both dorsal flanks of each mouse were injected subcutaneously with 0.3 ml Aff, Df, Rdf, or Crdf using a 1 ml syringe with a blunt infiltration cannula. Each recipient site was numbered and assigned to the four groups randomly. The grafts were injected in a hemispherical shape. Five animals in each group were sacrificed at 7, 15, 30, 60, and 90 days after injection. At the time of sacrifice, the grafts were harvested and carefully separated from surrounding tissue, and their volumes were measured. Each harvested sample was assessed histologically and immunohistochemically.

### Histologic Examination

Tissue samples were fixed in 4% paraformaldehyde (BD Biosciences), dehydrated, and embedded in paraffin. Tissue blocks were sectioned for staining with hematoxylin and eosin staining or Masson’s trichrome staining, examined under a BX51 microscope (Olympus, Tokyo, Japan), and imaged using a DP71 digital camera (Olympus). Total collagen content was reported as the percentage of the total tissue area positively stained with aniline blue, as determined using ImageJ (National Institutes of Health, Bethesda, MD, United States) software.

### Immunofluorescence Staining

Sample sections were stained with the following primary antibodies: rat anti-mouse Mac2 (1:200; Cedarlane Corp., Burlington, ON, Canada), rabbit anti-mouse CD206 (1:300; Abcam, Cambridge, MA, United States), goat anti-mouse perilipin-1 (1:200; Abcam), rat anti-mouse CD31 (1:200; Invitrogen, North Ryde, NSW, Australia), and rabbit anti-human alpha-smooth muscle actin (α-SMA) (1:200; Abcam). After washing, the samples were incubated with donkey anti-rat-555 immunoglobulin G (1:200; Abcam), donkey anti-goat-594 immunoglobulin G (1:200; Abcam) and donkey anti-rabbit-488 immunoglobulin G (1:200; Abcam) secondary antibodies. Nuclei were stained with DAPI (Sigma). The samples were examined under a TCS SP2 confocal microscope (Leica Microsystems GmbH, Wetzlar, Germany). Leica LAS AF software was used for images analysis.

### Enzyme-Linked Immunosorbent Assay (ELISA)

Enzyme-linked immunosorbent assay assays (Enzyme-linked Biotechnology, Shanghai, China) were performed to detect the levels of vascular endothelial growth factor (VEGF), basic fibroblast growth factor (bFGF), and transforming growth factor-β1 (TGFβ1) within Aff, Df or Rdf. Briefly, 1 ml of freshly prepared sample was mixed with 1 ml PBS and centrifuged at 1,200 *g* for 3 min. Extracted media were collected and subjected to sandwich ELISA assay.

### Quantitative Reverse Transcription Polymerase Chain Reaction (RT-PCR)

Fat tissue was excised, snap-frozen in liquid nitrogen, and stored at −80°C. Total RNA was extracted using TRIzol Reagent (Invitrogen) according to the manufacturer’s protocol. All primers shown in Table 1 designed for this study were determined through established GenBank sequences. The level of target gene was normalized to the housekeeping gene glyceraldehyde 3-phosphate dehydrogenase (GAPDH) and analyzed with the ABI PRISM 7500 Sequence Detection System with the SYBR Green PCR Master Mix (Sigma).

**TABLE 1:** Primer sequences for real-time RT-PCR.

Gene	Forward	Reverse
IL-10	5′ -AGTGGAGCAGGTGAAGA GTG-3′	5′ -TTCGGAGAGAGGTACAA ACG-3′
PPARγ	5′ -AGAACCTGCATCTCCAC CTT-3′	5′ -ACAGACTCGGCACTCAA TGG-3′
bFGF	5′ -AGCGGCTCTACTGCAAG AAC-3′	5′ -CCGTCCATCTTCCTTCA TAG-3′
LPL	5′ -AAGAAAACCCCAGCAAG GCA-3′	5′ -TAGCCCAGATTGTTACAG CGA-3′
C/EBPβ	5′ -CAAGCTGAGCGACGAGT ACA-3′	5′ -TCAGCTCCAGCACCTT GTG-3′
GAPDH	5′ -ACCCAGAAGACTGTGGA TGG-3′	5′ -CACATTGGGGGTAGGAA CAC-3′

### Statistical Analysis

Data were expressed as mean ± SEM and analyzed by repeated-measures analysis of variance. Independent *t*-tests were used to compare the two groups of mice at single time points, and one-way analysis of variance was used to compare the groups at all time points. A value of *p* < 0.05 was considered statistically significant.

## Results

### Removal of Adipose Components in Fat

**FIGURE 1:**
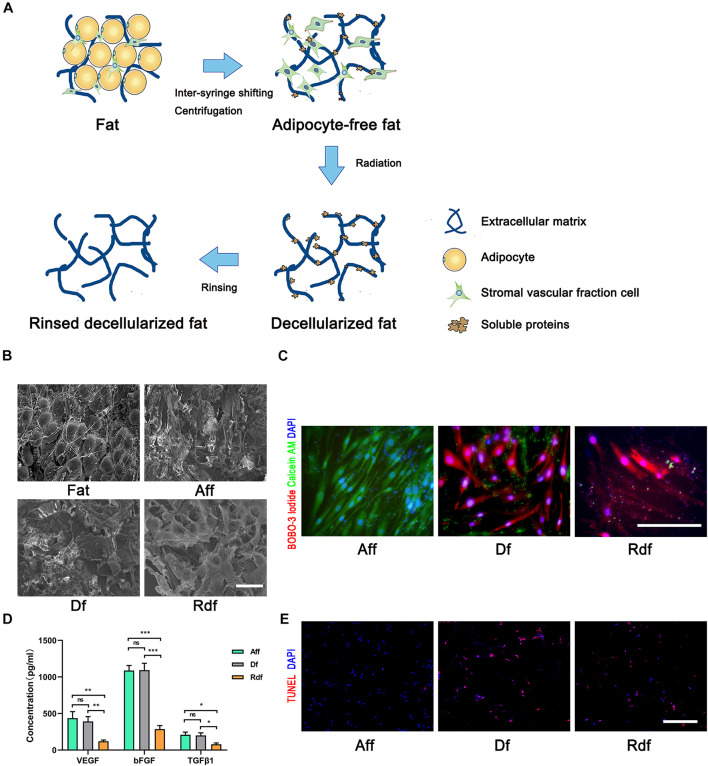
Removal of adipose components in fat. **(A)** Illustration showing stepwise procedures eliminating adipose components in fat. **(B)** Scanning electron microscopy views of fat, Aff, Df, and Rdf. **(C)** Live/Dead assay staining measuring SVF cell death of Aff, Df, and Rdf after 72 h of culture. **(D)** ELISA analyses levels of VEGF, bFGF and TNFβ1 in Rdf compared with Aff and Df (*p < 0.05, **p < 0.01, ***p < 0.001, ns = statistically non-significant). *n* = 3 independent experiments. **(E)** TUNEL staining to investigate cell apoptosis of Aff, Df, and Rdf on post-grafting day 1. Scale bar = 100 μm.

Stepwise mechanical processes were conducted to fat to obtain Aff, Df, Rdf (Figure 1A). Scanning electron microscopy revealed that most of the adipocytes were removed in Aff, Df, and Rdf (Figure 1B). Freshly prepared Df and Rdf were examined using Live/Dead and TUNEL staining to measure irradiation-induced cell death and apoptosis relative to that in fresh Aff. The results revealed that after isolation from grafts and culture for 72 h, SVF cells in Df and Rdf exhibited a fragmented and shrunken cell morphology with slow proliferation activity relative to Aff. Df and Rdf had significantly lower cell viability, with most of cells undergoing cell death after 72 h; by contrast, no cell death was observed in Aff (Figure 1C). The levels of VEGF, bFGF and TNFβ1 did not significantly differ between the Aff and Df groups, while significantly decreased in Rdf (Figure 1D). Due to the irradiation, more apoptotic cells were observed in the Df group and Rdf group than in the Aff group on day 1 after grafting (Figure 1E).

### Removal of Stromal Vascular Fraction and Microenvironment Impairs Graft Retention

**FIGURE 2:**
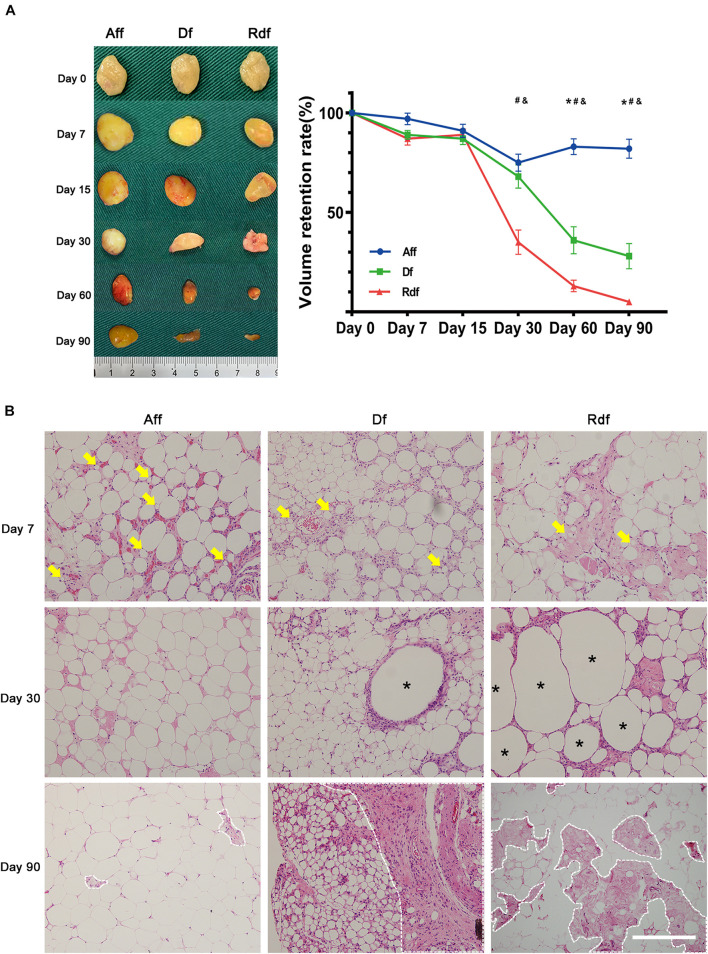
Removal of SVF and microenvironment impairs graft retention. **(A)** Graft appearance and retention rates over time. Quantification of graft volume, showing significantly higher retention rates in the Aff group versus Df (*p < 0.05) or versus Rdf (#*p* < 0.05) at later time points. The retention rate of Df was significantly higher than Rdf on days 30, 60, and 90 (&*p* < 0.05). *n* = 5 mice. **(B)** Histological changes in Aff, Df, and Rdf grafts on post-operative day 7, 30, and 90. Some small preadipocytes with multiple intracellular lipid droplets (arrows) were observed as early as day 7. Large amounts of necrotic tissue and large oil cysts (asterisks) were observed in the interior zone of Df and Rdf groups on day 30. Severe fibrosis was observed in the Df and Rdf groups on day 90 (dotted line). Scale bar = 100 μm.

In the next experiments, we harvested Aff, Df, and Rdf at different time points (Figure 2A). The Aff and Df grafts showed a similar appearance and texture from days 0 to 90, both covered with a thin, well-vascularized fibrous capsule. While Rdf grafts appeared to be less integrate after day 30 with a sharp decline in volume. The volumes of the Aff and Df grafts remained relatively constant before day 30 (Figure 2A, left panel). Quantification of graft volume indicated that the volume of Aff decreased over the first 30 days and maintained its volume afterward. The volume of Df and Rdf decreased markedly from days 15 to 90. On day 90, Aff had the highest retention rate (82 ± 7.1% vs. Df, 28 ± 6.3%; ^∗^*p* < 0.05; vs. Rdf, 5 ± 1.2%; #*p* < 0.05). The retention rate of Df was significantly higher than Rdf on day 90 (28 ± 6.3% vs. 5 ± 1.2%; &*p* < 0.05) (Figure 2A, right panel).

Histologic analysis showed that both the superficial and central areas of the Aff group contained large numbers of small preadipocytes (arrows), extensive well-vascularized connective tissue, and infiltrated cells on day 7 after transplantation (Figure 2B). By day 30, Aff developed large numbers of mature adipocytes showing a normal adipose structure. By contrast, although a few small preadipocytes appeared within peripheral area of the Df graft, some large oil cysts (asterisks) hadn’t been completely absorbed by day 30. Df exhibited large proportion of fibrotic area (dotted line) with intensive cell infiltration in the interior zone on day 90. As in the Rdf group, small-sized preadipocytes were barely found at the early stage after grafting. Large proportion of graft were occupied by necrotic tissue and large oil cysts on day 30. By day 90, the central areas of Rdf exhibited deconstructed adipose morphology with appearing of tissue debris, severe fibrosis and little cell infiltration. Quantification of the percentage of the fibrosis area showed significantly higher levels of fibrosis in the Df and Rdf group on day 90 (^∗^*p* < 0.05) (Supplementary Figure 1).

### Impaired Angiogenesis and Adipogenesis in Decellularized Fat and Rinsed Decellularized Fat

**FIGURE 3:**
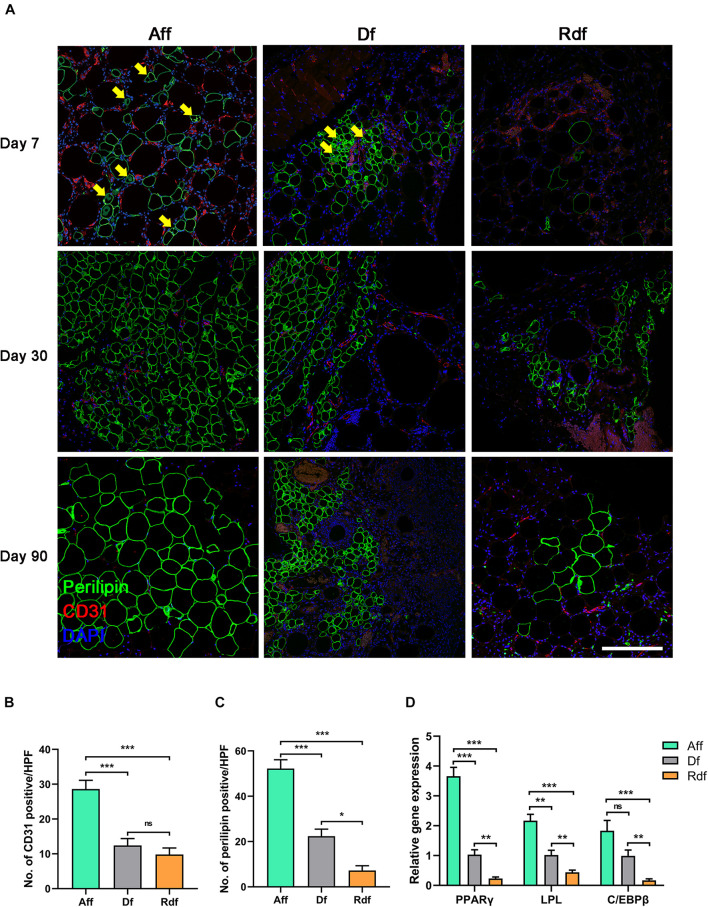
Impaired angiogenesis and adipogenesis in Df and Rdf. **(A)** Representative immunofluorescence staining of perilipin (green) and CD31 (red) in Aff, Df, and Rdf grafts on post-operative day 7, 30, and 90. Early-differentiated preadipocytes (arrows) were identified in Aff and Df on day 7. Quantification of **(B)** CD31-positive area and **(C)** perilipin-positive area in grafts on post-operative day 7 per high power field (*p < 0.05, ***p < 0.001, ns = statistically non-significant). **(D)** Relative transcription of PPARγ, LPL, and C/EBPβ (**p < 0.01, ***p < 0.001, ns = statistically non-significant). *n* = 3 independent experiments with 5 samples each group. Scale bar = 100 μm.

Immunostaining of CD31/perilipin revealed the earliest angiogenesis in Aff on day 7 (Figure 3A). At the time when angiogenesis was observed, the Aff graft did not exhibit an obvious necrotic area but instead arose plenty of early-differentiated preadipocytes (arrows) with a distinctive multiple perilipin + lipid droplet morphology. By day 30, Aff developed large number of small-sized immature adipocytes, which grew and matured by day 90. By contrast, limited vascular fractions were observed in Df and Rdf. In coordination with angiogenesis, Df exhibited limited adipogenesis with appearing of regional small adipocytes in the periphery. Central area remained perilipin- by day 90. Moreover, Rdf had the poorest adipogenesis with only a few perilipin + adipocytes displayed in the graft. Large area was CD31- and perilipin-. The mean number of vessels per high-power field (HPF) in Aff was significantly higher than that in the Df and Rdf group at post-operative day 7 (Aff, 28.6 ± 2.53 vs. Df, 12.4 ± 1.97 vs. Rdf, 9.8 ± 1.89, ^∗^*p* < 0.05) (Figure 3B). Quantification of adipocytes revealed that adipogenesis on day 7 was best in Aff, still prominent in Df, and weakest in Rdf (52.2 ± 3.91 vs. 22.4 ± 3.08 vs. 7.2 ± 2.13, ^∗^*p* < 0.05) (Figure 3C). Quantitative polymerase chain reaction (qPCR) analysis assay showed that adipogenic relative genes, peroxisome proliferator-activated receptor gamma (PPARγ), lipoprotein lipase (LPL), and CCAAT/enhancer-binding protein beta (C/EBPβ), of both Aff and Df groups were significantly higher than that in the Rdf group (^∗^*p* < 0.05). Compared with the Df group, the expression of PPARγ and LPL was significantly higher in the Aff group (^∗^*p* < 0.05) (Figure 3D).

### Adipose-Derived Stem Cell Supplementation Rescue Poor Adipogenesis and Reverse M1 Inflammation in Decellularized Fat

**FIGURE 4:**
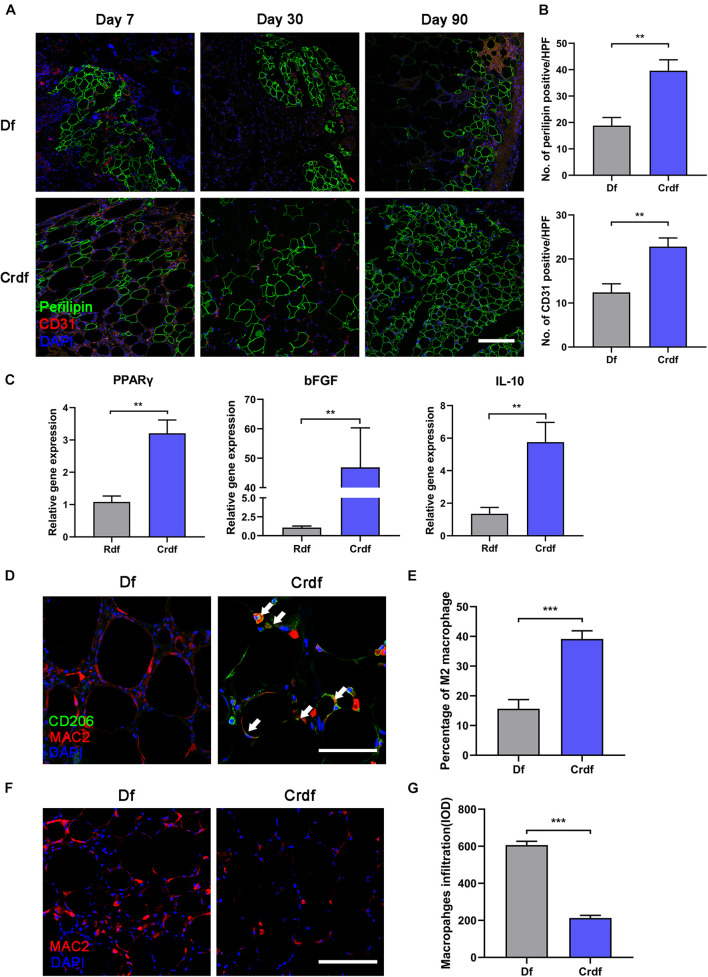
ASC supplementation rescue poor adipogenesis and reverse M1 inflammation in Df. **(A)** Representative immunofluorescence staining of perilipin (green) and CD31 (red) in Df and Crdf on post-operative day 7, 30, and 90. **(B)** Quantification of perilipin-positive area and CD31-positive area in grafts on post-operative day 30 per high power field. (**p < 0.01). **(C)** Relative transcription of PPARγ, bFGF, and IL-10 (**p < 0.01). **(D)** Representative immunofluorescence staining of CD206 (green) and Mac2 (red) in Df and Crdf on post-operative day 7. **(E)** Quantification of percentage of CD206 + /Mac2 + M2 macrophage in Mac2 + macrophage in grafts on post-operative day 7 per high power field (***p < 0.001). **(F)** Representative immunofluorescence staining of Mac2 (red) in Df and Crdf on post-operative day 90. **(G)** Quantification of Mac2 + macrophage infiltration in grafts on post-operative day 90 (***p < 0.001). *n* = 3 independent experiments with 5 samples each group. Scale bar = 100 μm.

To explore the regenerative ability of supplemented ASCs, CD31/perilipin immunofluorescent staining was applied to determine the angiogenesis and adipogenesis in Crdf versus Df (Figure 4A). With ASC supplementation, Crdf contained a few more CD31 + spots on day 7 compared with the Df group. By day 90, Crdf developed appreciable number of small-sized adipocytes scattered in both interior and periphery of the graft. By contrast, Df exhibited regional adipogenesis mostly at the periphery of the graft. Quantification of adipocytes indicated improved adipogenesis in the Crdf group compared with that in the Df group (Df, 18.8 ± 3.08 vs. Crdf, 39.6 ± 4.18, ^∗^*p* < 0.05) (Figure 4B). The mean number of vessels was significantly higher in Crdf than that in the Df group at post-operative day 7 (22.8 ± 1.98 vs. Df, 12.4 ± 1.97, ^∗^*p* < 0.05). To evaluate the adipogenic and angiogenic capacity of the supplemented ASCs, PPARγ and bFGF were assessed by quantitative RT-PCR. Results showed that PPARγ and bFGF mRNA expression was significantly higher in the Crdf group (^∗^*p* < 0.05). A higher level of interleukin-10 (IL-10) expression was also observed in the Crdf group (^∗^*p* < 0.05) (Figure 4C).

By day 7, immunofluorescence staining revealed that macrophages in the Crdf group tended to be M2-polarized (Mac2 + /CD206 +) (white arrow), whereas Mac2 + /CD206- M1 macrophages were predominant in Df grafts (Figure 4D). Quantification of the M2 macrophage indicated significantly higher percentage of M2 macrophage infiltration in Crdf than Df (39.13 ± 3.1 vs. Df 15.65 ± 2.72, ^∗^*p* < 0.05) (Figure 4E). By day 90, prolonged Mac2 + macrophage infiltration was observed in Df compared with Crdf (Figure 4F). Quantification of macrophage infiltration revealed that the number of macrophages in Df was significantly higher than that in Crdf (606.4 ± 20.98 vs. Df, 213.5 ± 14.22, ^∗^*p* < 0.05) (Figure 4G).

### Adipose-Derived Stem Cells in Cell-Recombinant Decellularized Fat Differentiate Into Vascular Endothelium

**FIGURE 5:**
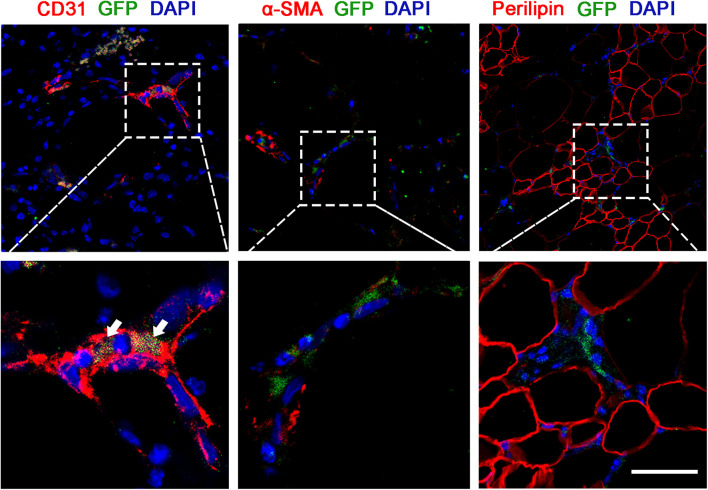
ASCs in Crdf differentiate into vascular endothelium. GFP fluorescence in conjunction with CD31, αSMA, and perilipin immunofluorescence were applied to identify the transformation of supplemented GFP + ASCs. Scale bar = 50 μm.

We examined the fluorescence signals of GFP-transfected ASCs in the Crdf group by immunofluorescence staining (Figure 5). Colocalization of GFP + ASCs with CD31 + endothelium was observed in the graft (white arrow). αSMA + pericytes were not colocalized with ASCs, indicating that ASCs were mainly involved in endothelium formation during vascularization. Similarly, no GFP + /perilipin + adipocytes were detected in the Crdf group, indicating that the ASCs had undergone minimal adipogenic differentiation.

## Discussion

Our previous study demonstrated that mechanical destruction and removal of adipocytes from the fat graft did not compromise the retention rate or the adipogenic potential (Zhang et al., 2018). To our surprise, the removal of adipocytes decreased the inflammatory level in the fat and enhanced adipogenesis (Zhang et al., 2018). Other studies revealed that the apoptosis of adipocytes is conductive to induce inflammatory infiltration and caused oil cysts after fat grafting (Lancaster and Langley, 2014; Nishimoto et al., 2016). These results suggested that adipocytes are not the key element for adipose regeneration after fat grafting, and the other components should make great contribution to adipogenesis. This is the first study of the regenerative ability of ASCs, ECM, and microenvironment exclusively after transplantation.

The results suggested that the long-term retention rate was significantly higher for Aff and Df grafts than for Rdf grafts (82 ± 7.1% vs. Df, 28 ± 6.3%; ^∗^*p* < 0.05; vs. Rdf, 5 ± 1.2%; #*p* < 0.05). At the early stage of transplantation, adipogenesis was observed in all groups except the Rdf group. Initial adipose regeneration developed further in Aff and Crdf grafts, but not in Df grafts. At 90 days post-grafting, Df exhibited large amounts of necrotic tissue and large oil cysts in the interior zone. The regenerative mode of Df was characterized by impaired angiogenesis and M1 macrophage infiltration, whereas M2 macrophage infiltration and active angiogenesis were observed in Aff and Crdf. In addition, ASCs directly participated in vessel formation and exhibited an endothelial phenotype.

Previous studies demonstrated that decellularized adipose matrix (DAM) has the potential to induce adipogenesis (Flynn, 2010; Turner et al., 2012; Trivanovic et al., 2018). While transplantation of DAM leads to severe fibrotic tissue morphology, with pronounced M1 macrophage infiltration (Turner et al., 2012). Moreover, another study suggests that the adipose induction potential of DAM can be influenced by the preparation methods (Brown et al., 2011). Because the preparation of DAM requires full clearance of antigens, the adipose tissue in this study underwent multiple and prolonged procedures that may influence the preservation of soluble protein. Soluble growth factors, which are the important constituents of microenvironment, had a positive role in promoting long-term preservation of DAM (Chun et al., 2019). Using supplemental bFGF, Zhang et al. (2016) reported fat regeneration and long-term retention of DAM 12 weeks after transplantation. To minimize damage to the adipose ECM and soluble microenvironment, we applied a mechanical process and radiation to remove all viable cellular components, thereby preserving the native ECM and microenvironment. As with DAM, Df could also induce adipogenesis with the appearance of scattered perilipin + immature adipocytes at the early stage of grafting. While further removing soluble protein, adipogenesis was barely found in Rdf transplantation, indicating that retention of the abundant extracellular microenvironment within the adipose ECM may be the key to inducing fat regeneration.

Many studies had shown that without adipose-inductive scaffolds and microenvironment, transplantation of ASCs does not generate adipose tissue (Acil et al., 2014; Kurzyk et al., 2019). However, we found that they played an important role in activating the adipoinduction potential of the ECM and microenvironment. After supplementation of Df with ASCs, Crdf had higher long-term retention rate and greater tissue integrity than Df. The number of CD31 + endothelial cells was significantly higher for Crdf than for the Df group indicating improved angiogenesis.

Moreover, Crdf induced transient M2-polarized macrophages infiltration, in contrast to the chronic M1 macrophages in the Df group. Previous studies had shown that rapid inflammatory responses could activate chemotaxis of host-derived mesenchymal stem cells and promote angiogenesis in the early stage of grafting (Zhan and Lu, 2017; Cai et al., 2018). While persistent high levels of macrophage infiltration inhibit adipogenesis and cause severe fibrosis (Cai et al., 2017a,b). M2 polarization of macrophages help decreases inflammation and promotes tissue remodeling via secretion of anti-inflammatory and angiogenic cytokines (Jetten et al., 2014). Increasing the proportion of M2-type macrophages can facilitate better long-term retention and tissue texture in fat grafting (Dong et al., 2013). Collectively, these results suggest that ASCs can decrease fibrosis and create a better microenvironment for adipogenesis.

GFP-ASC tracing revealed that ASCs presented in the stromal area and participated mainly in formation of vascular endothelium rather than adipocytes or pericytes. In a previous study, adipocytes were derived from the graft (Doi et al., 2015), and another study showed that donor ASCs could develop into newly differentiated adipocytes (Hong et al., 2018), which seems to contradict our results. Those authors used a CAL model to identify the role of ASCs, whereas we used Df + ASC model. Different cellular components were used in these models, which might explain the differences in our results. We removed all SVF cells from the transplanted fat, whereas Hong’s model preserved native SVF cells. Both studies confirmed that ASCs could develop into endothelial cells and may incorporate the host blood supply (Laschke et al., 2009), thereby accelerating the revascularization of the graft. These findings suggest that ASCs serve as vascular endothelium to extend the range of adipogenesis within the graft; however, further studies are required to determine the detailed interactions between grafted ASCs and neonatal adipose precursors.

**FIGURE 6:**
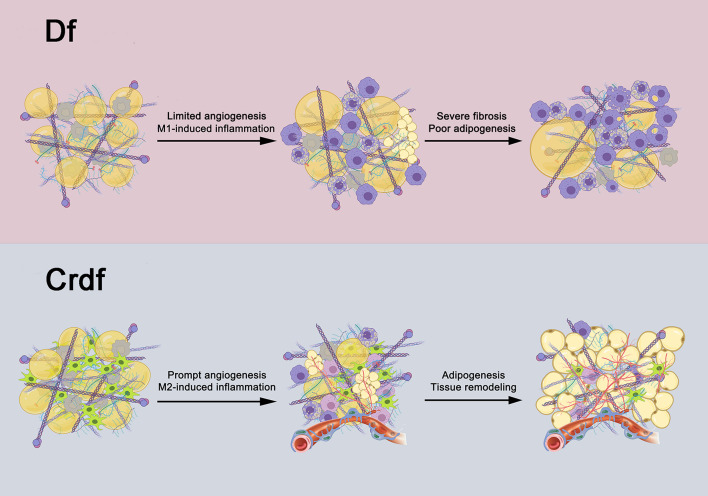
Potential role of ASCs in fat regeneration. After all adipocytes and SVF cells within the graft are removed, adipogenesis can be induced by the transplanted adipose ECM and the microenvironment in Df. However, the sustained presence of M1 inflammation and slow angiogenesis leads to poor adipogenesis and severe fibrosis. Supplementation with ASCs in Df can drive M1 macrophages into M2 polarization as well as develop into endothelial cells of vessels, which promote inflammation resolution and enhance angiogenesis, collectively contribute to mature adipogenesis.

Figure 6 summarizes the adipogenesis outcomes with or without supplemented ASCs of Df based on the results of this study. Removing all cellular components through mechanical processes and irradiation, the adipose ECM and microenvironment are capable of initiating adipogenesis. However, the sustained predominance of M1 inflammation and limited angiogenesis led to poor adipogenesis and severe fibrosis in the graft. By shifting to an M2-dominated milieu and differentiating into vascular endothelium, ASCs promote angiogenesis and adipogenesis, thereby facilitating complete reconstruction of adipose tissue.

In addition, it is worth mentioning that the Aff, which contained SVF instead of ASCs as its cellular component, had a higher long-term retention rate than Crdf (data not presented). This indicated that other cells in SVF may also contribute to adipose reconstruction. Many studies have reported that regulatory T-cells (Treg) are involved in resolving inflammation resolution during tissue repair by controlling neutrophils, inducing macrophage polarization, and regulating helper T-cells (Weirather et al., 2014; Dombrowski et al., 2017). Further characterization of the cellular components involved in adipose regeneration is required to enabling sophisticated adipose induction strategy.

## Conclusion

This preliminary animal study demonstrated that grafting of adipose ECM and microenvironment have the capacity to stimulate early adipogenesis *in vivo*. Whereas insufficient graft vascularization and chronic M1 inflammation lead to fibrosis and necrosis outcome. Further removal of microenvironment lost the capacity of inducing adipogenesis. By directly differentiating into endothelial cells and regulating M2 polarization of macrophage, supplementation of ASCs increased the long-term retention rate and had a greater tissue integrity after transplantation.

## Data Availability Statement

The original contributions presented in the study are included in the article/Supplementary Material, further inquiries can be directed to the corresponding authors.

## Ethics Statement

The animal study was reviewed and approved by the Nanfang Hospital Institutional Animal Care and Use Committee.

## Author Contributions

YZ and JiaG: conception and design, financial support, and final approval of the manuscript. WJ and JC: conception and design, manuscript writing, assembly of data, and data analysis and interpretation. JinG and YL: assembly of data. FL and JM: data analysis and interpretation. All authors contributed to the article and approved the submitted version.

## Conflict of Interest

The authors declare that the research was conducted in the absence of any commercial or financial relationships that could be construed as a potential conflict of interest.

## Publisher’s Note

All claims expressed in this article are solely those of the authors and do not necessarily represent those of their affiliated organizations, or those of the publisher, the editors and the reviewers. Any product that may be evaluated in this article, or claim that may be made by its manufacturer, is not guaranteed or endorsed by the publisher.

## Supplementary Material

Supplementary Figure 1 Masson’s trichrome staining of Aff, Df, and Rdf grafts. **(A)** On day 90, thick fibrous tissue was observed in Df and Rdf grafts. By contrast, Aff grafts showed reduced collagen accumulation and normal adipose tissue structure. **(B)** Quantification of fibrosis areas in the grafts, showing that the levels of fibrosis were significantly higher in the Df and Rdf group on day 90 (*p* < 0.05). *n* = 5 mice. Scale bar = 100 μm.
