# Supplementary figures and images for: Characterized the Adipogenic Capacity of Adipose-Derived Stem Cell, Extracellular Matrix, and Microenvironment With Fat Components Grafting
Source: Front Cell Dev Biol. 2021 Sep 20;9:723057. doi: 10.3389/fcell.2021.723057 (PMC8489879; doi:10.3389/fcell.2021.723057)

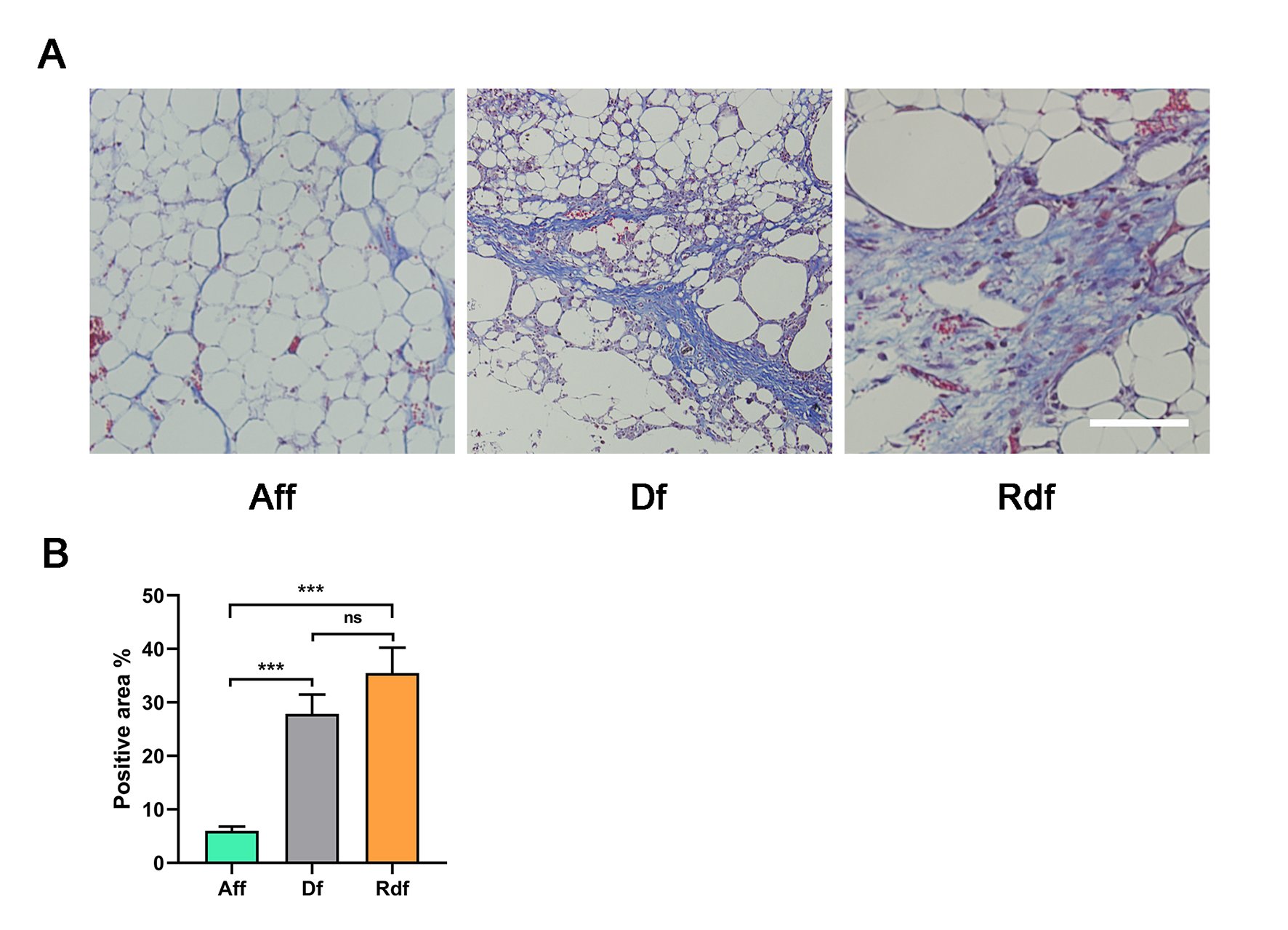

Supplement: Supplementary Figure 1 — Masson’s trichrome staining of Aff, Df, and Rdf grafts. (A) On day 90, thick fibrous tissue was observed in Df and Rdf grafts. By contrast, Aff grafts showed reduced collagen accumulation and normal adipose tissue structure. (B) Quantification of fibrosis areas in the grafts, showing that the levels of fibrosis were significantly higher in the Df and Rdf group on day 90 (p < 0.05). n = 5 mice. Scale bar = 100 μm. [file Image_1.TIF]
